# Polystyrene Microplastics of Varying Sizes and Shapes Induce Distinct Redox and Mitochondrial Stress Responses in a Caco-2 Monolayer

**DOI:** 10.3390/antiox12030739

**Published:** 2023-03-17

**Authors:** Nelly D. Saenen, Margo S. Witters, Inneke Hantoro, Inés Tejeda, Anitha Ethirajan, Frank Van Belleghem, Karen Smeets

**Affiliations:** 1 Centre for Environmental Sciences, Hasselt University, Agoralaan Building D, 3590 Diepenbeek, Belgium; nelly.saenen@uhasselt.be (N.D.S.); margo.witters@uhasselt.be (M.S.W.); ines.tejeda@uhasselt.be (I.T.); frank.vanbelleghem@uhasselt.be (F.V.B.); 2 Food Technology Department, Soegijapranata Catholic University, Jl. Pawiyatan Luhur IV/1, Bendan Duwur, Semarang 50234, Indonesia; inneke.hantora@unika.ac.id; 3 Nano-Biophysics and Soft Matter Interfaces Group, Institute for Materials Research, Hasselt University, Wetenschapspark 1, 3590 Diepenbeek, Belgium; anitha.ethirajan@uhasselt.be; 4 IMEC, Associated Lab IMOMEC, Wetenschapspark 1, 3590 Diepenbeek, Belgium; 5 Department of Environmental Sciences, Open Universiteit, Valkenburgerweg 177, 6419 AT Heerlen, The Netherlands

**Keywords:** microplastics, in vitro, uptake, oxidative stress, mitochondria, spheres, fibres, fragments

## Abstract

Currently, we lack crucial knowledge on how the physicochemical properties of particles affect cellular health, resulting in an important gap in our understanding of the human toxicity of microplastics (MPs). Our aim was to evaluate the impact of the size and the shape of MPs on uptake and the intracellular effects in a human epithelial colorectal adenocarcinoma (Caco-2) cell line. Spherical (200 nm and 2 µm) and fibre-/fragment-shaped (8.9 ± 10.1 µm by 1.14 ± 0.97 µm) polystyrene microplastics (PS-MPs) were used to study their uptake and the potential to induce redox and mitochondrial stress responses after 24 h of exposure. We demonstrated the cellular uptake of both spherical and fibre-/fragment-shaped MPs in a size-dependent manner. In response to 2 µm spheres, we observed differential expressions of redox-related genes, including *HMOX1*, *CAT*, and *GPX1*. All PS-MPs decreased the intracellular H_2_O_2_ levels, which can be attributed to mitochondrial stress responses, such as increased mitochondrial DNA content, footprint, and morphology. Altogether, we demonstrated uptakes and changes in redox and mitochondrial parameters for all PS-MPs, with the 200 nm spheres showing the most profound effects. This suggests that the induction of defensive responses in Caco-2 cells mainly correlates with the number of particles taken up.

## 1. Introduction

Plastics are ubiquitous due to their ease of use, mechanical properties, and low cost [1]. The annual global production increased nearly 300-fold, up to 368 million metric tons in 2019. Most of the plastics produced are used in (single-use) packaging, building and construction, and the automotive industry [2,3,4], of which 8 million tons are estimated to end up in the oceans as mismanaged plastic waste [5]. As a result, plastics account for 80% of marine debris and pose a major environmental risk. In addition to the visible harmful consequences such as the entanglement of marine biota by plastics, the presence of micro- and nanoplastics in the soil, air, and water raises many concerns about their impact on the environment and human health.

Microplastics (MPs) are commonly defined as plastic particles smaller than 5 mm originating from a variety of sources including cosmetics, clothing, and industrial processes. MPs are classified as either primary MPs, which are intentionally manufactured for industrial or household applications including facial scrubs and toothpaste [6], or secondary MPs which are formed when larger plastics are weathered by photochemical (UV radiation), mechanical (wave action), or biodegradation processes (microorganisms). These secondary MPs are the main source of MP pollution in the environment and have the potential to degrade to nanoplastics (NPs, <0.1 µm) [6,7]. Aside from different sizes, micro- and nanoplastics exist in a variety of shapes and chemical compositions. Polyethylene (PE), polypropylene (PP), and polystyrene (PS) are the most abundant polymer types [8], accounting for about 33% of marine litter, along with other media such as sediments, soils, and sewages [9]. Fibres, fragments, and films are the main forms found in environmental samples, including drinking water and freshwater [10].

When MPs are deposited in the environment, they can be ingested by marine organisms from different trophic levels, entering our food web. In addition to seafood and drinking water, MPs have also been found in food products and beverages, including honey and beer [11,12]. Ingestion is, therefore, one of the main routes of human exposure, as evidenced by the presence of MPs in human faeces [13].

Although recent studies have discovered MPs in human blood [14] and in placental tissue [15], adequate hazard and risk assessments are still lacking. The multidimensionality of MPs, as reflected in their diverse physicochemical properties due to a heterogeneity of sources, transport properties, pollution loading, and uptake by a range of species, poses a major challenge for assessing their risks [16]. To date, studies of MPs in human model systems have mainly focused on polystyrene spheres within a narrow size range. Adverse effects of MPs exposure on the intestinal system were shown in vivo. Choi et al. (2021) reported that the oral administration of 0.5 µm PS spheres for 2 weeks induced constipation by altering the physiological and mechanical functioning of the mid colon in ICR mice [17]. Other common effects are inflammation [18,19,20,21] and oxidative stress [21]. It is often hypothesized that MPs’ size is important for cellular uptake and underlying effects. According to a recent review, cells can take up MPs as large as 10 µm [22]. For example, Stock et al. (2019) showed that polystyrene spheres of 1 µm, 4 µm, and 10 µm were taken up by human epithelial colorectal adenocarcinoma cells (Caco-2) [18]. The degree of cellular uptake, particle size, and cell model underlie the variability in biological effects [22]. To overcome these contradictory findings, MPs research should invest in the study of multi-parameter biological effects and investigate multiple MPs’ properties, such as size and shape.

The variability of MPs’ physicochemical properties and the associated complexity in the interaction with the human biological systems require an in-depth investigation at the fundamental level into carefully chosen model systems in order to elucidate MPs’ fate [23]. A human Caco-2 cell line was used in the current study, because (i) ingestion is one of the main routes of MPs exposure, (ii) a simple in vitro model system is required to be able to link physicochemical properties with biological effects, and (iii) in vitro research is in line with the 3Rs principle and the REACH guidelines is needed to reduce animal experiments [24,25]. The specific aim of this study was to investigate the effect of both MPs size and shape on uptake and their potential intracellular effects in Caco-2 cells. Because of particle interference, multiple assays were used for the different stress responses. The integration of the physicochemical properties of MPs with their effects at multiple biological levels provides key information for accurate future hazard profiling of MPs.

## 2. Materials and Methods

### 2.1. Materials

Commercially available red fluorescent and non-fluorescent polystyrene beads, functionalized with carboxylic surface groups (-COOH), were purchased from Magsphere Inc. (Pasadena, CA, USA). In total, 2 particle diameters were used in this study: 200 nm and 2 µm. The material was supplied by the manufacturer in an aqueous suspension of 2.5% *w*/*v* for the fluorescent particles and 10% *w*/*v* for the non-fluorescent particles. The particle information of the manufacturer is provided in Table 1. Stock suspensions of 1 mg/mL were prepared in MilliQ, sterilised with gamma-irradiation for 20 min at 30 Gy, and stored in the dark at 4 °C without further purification.

### 2.2. Synthesis of PS Microfibres and Fragments using a Centrifugal Spinning Setup

For the synthesis of PS microfibres and fragments (PSMFs), polystyrene granules (Mw = 190 kg/mol) and tetrahydrofuran (THF, AnalaR NORMAPUR) were purchased from VWR Chemicals (Oud-Heverlee, Belgium) and used without further purification. The polymer solutions were prepared by slowly adding the polymer to the solvent while continuously magnetically stirring for 24 h in hermetically sealed vials to ensure a homogeneous mixture. A stock suspension of 12.5 wt% was prepared. The polystyrene fibres were then synthesised using a home-built centrifugal spinning setup (kindly provided by the group of Prof. Naveen Reddy, IMO-IMOMEC, Hasselt University, Belgium) [26]. Briefly, 4 mL of the stock suspension was added at a constant flow rate of 1 mL/min via a syringe pump to the rotating spinneret equipped with 0.6 mm aluminium nozzles. The polystyrene fibres were produced at a rotation speed of 1252× *g* at room temperature (22 °C) and a relative humidity of 40%. The fibres were then captured between collector poles 12 cm away from the nozzle tips, collected, and stored in a sealed glass container at room temperature.

Thereafter, the fibres were ground twice in a small amount of liquid nitrogen using a mortar and pestle, collected in MilliQ with 0.01% Tween 80 (Sigma-Aldrich, Darmstadt, Germany) and passed through a nylon mesh filter (41 µm pore size, Merck Millipore, Germany) using vacuum filtration. The supernatant was then filtered through a polycarbonate filter (10 µm pore size) (Whatman Nuclepore Polycarbonate Track-Etched Membranes, Sigma-Aldrich, Darmstadt, Germany). After drying the PSMF at 65 °C, a final mass of 5 mg was obtained. Then, half of the PSMF was stained with iDye Poly Pink (Jacquard, IDYE-456, Healdsburg, CA, USA), while the rest was suspended in phosphate-buffered saline (PBS, Gibco, Fisher Scientific, Brussels, Belgium). For the staining, dry PSMFs (2.5 mg of PSMF per mL) were added to 100 mg/mL of iDye Poly Pink solution. This solution was heated at 70 °C for 2 h in the dark. Next, the stained PSMF were poured through a polycarbonate filter (10 µm pore size) using vacuum filtration and rinsed with PBS. Thereafter, they were dried at 65 °C in the dark, weighed, and suspended in PBS. After 20 min of gamma-irradiation at 30 Gy, all stock suspensions of PSMF were stored at 4 °C. To avoid external contamination, all procedures were performed in a fume hood without the use of plastic consumables. Based on the limited mass of PSMFs available, a selection of cellular and molecular parameters was measured.

### 2.3. Physical Characterisation of PS-MPs

For the commercially available microbeads, transmission electron microscopy (TEM) samples were prepared by placing the pioloform-coated 150 mesh copper grid on a drop of the particle suspension (100 µg/mL) for 10 min. The grids were pre-treated with alcian blue to render a positive charge. After incubation, the TEM grid was removed from the droplet and dried with filter paper from below to avoid a loss of the particulate material. The particle size distribution was measured by quantitative TEM analysis [27]. Briefly, a set of at least ten TEM micrographs was randomly recorded from various regions of the TEM grid using brightfield TEM mode employing a JEM-1400 Flash (JEOL, Tokyo, Japan), equipped with a bottom mount 20 MP XAROSA CMOS camera (EMSIS, Münster, Germany), operating at 80 kV. Magnifications in the range of 500 to 5000 times were selected. Analyses were carried out for at least 500 particles, allowing for the reliable determination of the particle size distributions, with the NanoDefine ParticleSizer software plugin in ImageJ 2.9.0/1.53t (NanoDefine 2016). The distributions of the minimum Feret diameter (Feret min), the aspect ratio, and the shape were determined. The size and size distributions of the different colloidal suspensions were studied using dynamic light scattering (DLS) (Zetasizer Ultra-Red, Malvern Panalytical, Malvern, UK) for the PS 200 nm spheres, and static light scattering (SLS) (Mastersizer 3000, Malvern Panalytical, Malvern, UK) for the PS 2 µm spheres. The zeta-potential was measured in diluted stock suspensions using 1 mM potassium chloride (KCl) (Thermo Scientific Chemicals, Fisher Scientific, Brussels, Belgium).

The PSMFs were characterised using a Nikon Eclipse Ts2 stereomicroscope (Nikon Europe, Amstelveen, The Netherlands). A set of ten micrographs were randomly recorded in brightfield mode, equipped with a Nikon DS-Fi3 camera operating at 40× magnification. Analyses were performed for at least 350 particles using ImageJ 2.9.0/1.53t software to determine the particle diameter and length.

### 2.4. Caco-2 Cell Culturing Protocol and Experimental Set-Up

Adherent human epithelial colorectal adenocarcinoma cells (Caco-2, ATCC^®^ HTB-37TM, LGC Standards, Wesel, Germany) were cultured and maintained in 75 cm^2^ cell culture flasks (Greiner, Sigma-Aldrich, Darmstadt, Germany) containing Dulbecco’s Modified Eagle Medium (DMEM, Gibco, Fisher Scientific, Brussels, Belgium), supplemented with 10% heat inactivated foetal bovine serum (FBS, Sigma-Aldrich, Darmstadt, Germany), 1% penicillin/streptomycin (Gibco, Fisher Scientific, Brussels, Belgium), and 1% non-essential amino acids (MEM-NEAA, Gibco, Fisher Scientific, Brussels, Belgium). The cells were cultivated at 37 °C and 5% CO_2_, and the medium was refreshed twice a week. For the experiments, cells with passage numbers between 30 and 40 were used to establish a confluent Caco-2 monolayer (non-synchronised cells; incubation for 5 days at 37 °C and 5% CO_2_; medium refreshed once). The exposure conditions selected for the experiments (0, 10, and 100 µg/mL of the PS-MPs) were based on preliminary concentration range-finding experiments (0 to 500 µg/mL of PS-MPs) and on the ability to compare the findings with literature references.

### 2.5. Quantification of PS-MPs Associated with Caco-2 Cells

The association (=attachment and/or uptake) of MPs was quantitatively assessed with fluorescence-activated cell sorting (FACS, FACSCalibur, BD BioSciences, San Jose, CA, USA). The cells were seeded in a 24-well plate at a density of 3 × 10^4^ cells/well and incubated at 37 °C and 5% CO_2_ for 5 days to establish a confluent Caco-2 monolayer. The monolayer was then exposed for 24 h to 0 and 100 µg/mL PS200RF, PS02RF, and PSMF (stained with Idye Pink) in DMEM without FBS. After detaching with 0.05% trypsin (Gibco, Fisher Scientific, Brussels, Belgium), the cells were washed twice in FACS buffer [PBS (Gibco, Fisher Scientific, Brussels, Belgium) + 2% FBS]. The cells were collected by centrifugation at 300× *g* for 5 min between each step. Afterwards, the cells were fixed in 4% paraformaldehyde (Sigma-Aldrich, Darmstadt, Germany) for 10 min at RT, and washed again in FACS buffer. A total of 10,000 gated cells were measured with FACS. The percentage of cells associated with fluorescent PS-MPs was determined by plotting the fluorescence (FL2-H) versus the cell number. The percentages were corrected for auto-fluorescence by subtracting the control values.

### 2.6. Quantification of PS-MPs Uptake in the Caco-2 Monolayer

Immunofluorescence stains were used to determine the qualitative uptake of the fluorescent MPs using confocal laser scanning microscopy (CLSM, LSM 900, Zeiss, Zaventem, Belgium), equipped with high power diode lasers at 405, 488, 514, and 633 nm, and mounted on an inverted laser-scanning microscope Axio Observer Z1/7, Zeiss, Zaventem, Belgium). Briefly, the cells were seeded at a density of 3 × 10^4^ cells/24-well on ethanol-sterilized coverslips and cultured for 5 days to generate a confluent Caco-2 monolayer. After exposure to 100 µg/mL PS200RF, PS02RF, and PSMF (stained with Idye Pink) for 24 h in DMEM without FBS, the cells were washed twice with the PBS (+Ca^2+^, +Mg^2+^, 37 °C) (Gibco, Fisher Scientific, Brussels, Belgium) and stained with 25 µM CellMask Green Plasma Membrane Stain (Ex/Em: 535/522, Invitrogen, Fisher Scientific, Brussels, Belgium) in phenol-free DMEM medium (Gibco, Fisher Scientific, Brussels, Belgium) for 40 min at 37 °C and 5% CO_2_. Next, the cells were washed twice in PBS (+Ca^2+^, +Mg^2+^, 37 °C) and fixed in 4% formaldehyde (Sigma-Aldrich, Darmstadt, Germany) for 10 min at room temperature (RT). After washing 3 times with PBS (+Ca^2+^, +Mg^2+^, 37 °C), the cells were counterstained with 1 µM Hoechst 33342 (Ex/Em: 350/455, Invitrogen, Fisher Scientific, Brussels, Belgium) for 10 min at RT. Finally, after three washings in PBS (+Ca^2+^, +Mg^2+^, 37 °C), the coverslip with cells was mounted onto a glass microslide using Shandon Immu-mount (Fisher Scientific, Brussels, Belgium) and allowed to dry at 4 °C for 24 h. To distinguish between PS-MPs attached to the cell surface and PS-MPs taken up by cells, the XYZ acquisition mode was used (ZEN blue 3.1 software, LSM990, Zeiss, Zaventem, Belgium). For each condition, 3 z-stack images were scanned manually at 40× magnification for the presence of PS-MPs inside the cells (Ex/Em: 505–545/560–630). A semi-quantitative analysis was carried out by manually selecting images that included the entire thickness of the cell layer but did not include the particles attached to the cell boundary. Then, the number of PS-MPs within the entire cell layer, as well as the total number of cells in the scanned image, were counted. The results were presented as the number of particles per ten cells. The surface covered by PS-MPs inside the cells was calculated by the following formula: [(number of PS-MPs inside cells of the scanned image × 4πr^2^)/(number of cells within scanned image)]. The results were presented as the surface (nm^2^) PS-MPs per 10 cells.

### 2.7. Measurement of Cytotoxicity and Redox and Mitochondrial Stress Responses

#### 2.7.1. Assessment of Cell Viability

Several assays were used to assess the effects of PS-MPs on the cell viability. First, the metabolic activity was measured using the 3-(4,5-dimethylthiazol-2-yl)-2,5-diphenyltetrazolium bromide) tetrazolium reduction (MTT) cell proliferation assay (ATCC^®^ 30–1010 K, LGC Standards, Wesel, Germany. Briefly, 6 × 10^4^ Caco-2 cells were seeded in a 96-well clear culture plate and grown in DMEM medium with FBS to a confluent monolayer. Next, the monolayer was exposed to 0, 10, and 100 µg/mL of PS200RF, PS02RF, and PSMF for 24 h in DMEM medium without FBS at 37 °C and 5% CO_2_. After exposure, 10 µL of MTT reagent was added to the suspension and incubated at 37 °C and 5% CO_2_ for 2–4 h. The incubation time was dependent on the presence of an intracellular punctate purple precipitate, which was visualized periodically using a Nikon Eclipse Ts2 stereomicroscope (Nikon Europe, Amstelveen, The Netherlands). Then, 100 µL of detergent reagent was added and the plate was left at room temperature overnight. Thereafter, the absorbance at 570 nm was measured using a microplate reader (Fluostar Omega, BMG Labtech, Champigny-sur-Marne, France).

Second, the membrane integrity was assessed using the CyQUANT^TM^ LDH Cytotoxicity Assay Kit according to the manufacturer’s instructions (Fisher Scientific, Brussels, Belgium). Briefly, Caco-2 cells were seeded at a density of 6 × 10^4^ cells in a 24-well culture plate and cultured for 5 days to obtain a confluent monolayer. The cells were exposed to 0, 10, and 100 µg/mL PS200RF, PS02RF, and PSMF for 24 h in DMEM medium without FBS. Next, 45 min before the end of exposure, 100 µL of 10× CyQUANT^TM^ lysis buffer or MilliQ was added to the control cells to serve as a control of the maximum lactate dehydrogenase (LDH) activity and a control of the spontaneous LDH activity, respectively. After exposure, 50 µL of the cell medium was transferred to a clear 96-well plate. After adding a reaction mixture for 30 min at RT in the dark, the stop solution was added to stop the reaction and the absorbance of LDH release was measured using a microplate reader (Fluostar Omega, BMG Labtech, Champigny-sur-Marne, France) at 490 nm and 680 nm absorbance. The following formula was used to calculate the percentage cytotoxicity: [(Particle-treated LDH activity – Spontaneous LDH activity)/(Maximum LDH activity – Spontaneous LDH activity)] × 100. 

Third, the 5-carboxyfluorescein diacetate—acetoxymethyl ester (5-CFDA-AM) fluorometric assay (Invitrogen, Fisher Scientific, Brussels, Belgium) was used to measure a combination of the esterase enzymatic activity and the membrane integrity in a Caco-2 confluent monolayer after exposure to 0, 10, and 100 µg/mL PS200NF and PS02NF. Briefly, Caco-2 cells were seeded at 5 × 10^3^ cells/96-well culture plate in DMEM medium with FBS and monitored until a confluent monolayer was reached. After 24 h of exposure in DMEM with FBS, the cells were washed with PBS and incubated with 0.1% 5-CFDA-AM for 30 min (37 °C, 5% CO_2_, dark). Finally, the fluorescence intensity (Ex: 485/20 nm/Em: 528/20 nm) was measured using a microplate reader (Fluostar Omega, BMG Labtech, Ortenberg, Germany). 

For all cell viability assays, the possible optical interference was checked by assessing the absorbance/fluorescence of PS200RF, PS200NF, PS02RF, PS02NF, and PSMF in the same experimental setups without cells. If optical interference was observed, the values obtained were used as blanks.

#### 2.7.2. Transcriptional Analysis with RT-qPCR

We measured the gene expression of a selection of oxidative stress-related genes (Table 2) using a quantitative real-time polymerase chain reaction (RT-qPCR) to evaluate the effects of PS-MPs exposure on molecular redox signatures. Caco-2 cells were seeded at 6 × 10^4^ cells/24-well and grown to a confluent monolayer. After exposure to 0 and 100 µg/mL PS200RF, PS02RF, and PSMF for 24 h in DMEM medium without FBS, the cells were washed in PBS, trypsinised, resuspended in DMEM medium with FBS and pelleted by centrifugation (300× *g*). After removing the supernatant, the cells were immediately frozen in liquid nitrogen and stored at −80 °C for further processing. The total RNA was isolated from the cells by phenol-chloroform extraction. The purity (260/280 = ratio of sample absorbance at 260 and 280 nm; 260/230 = ratio of sample absorbance at 260 and 230 nm) and the concentration of the sample (absorbance at 260 nm) were determined using Nanodrop spectrophotometer (ND-1000, Fisher Scientific, Brussels, Belgium). After TurboDNase treatment (Turbo DNA-free kit, Invitrogen, Fisher Scientific, Brussels, Belgium), RNA was converted to cDNA using the Superscript III First-Strand Synthesis Supermix (Invitrogen, Fisher Scientific, Brussels, Belgium) according to the manufacturer’s instructions. The gene expression was measured using the 384-well format of the QuantStudio 5 RT-qPCR system (Applied Biosystems, Fisher Scientific, Brussels, Belgium). A mastermix (7.5 µL) containing Fast SYBR Green PCR Master Mix (Applied Biosystems, Fisher Scientific, Brussels, Belgium), 0.3 mM forward and 0.3 mM reverse primer, and RNAse free water, together with 2.5 µL of 10 ng/µL cDNA, were added to each well. The following program was initiated on the Quantstudio 5 RT-qPCR system (Fisher Scientific, Brussels, Belgium): 95 °C for 20 s, 40 cycles of 95 °C for 1 s, and 60 °C for 20 s. The 2^–∆∆Ct^ method, normalized for three reference genes (Table 2), was applied to calculate the fold gene expression relative to the control.

#### 2.7.3. Measurement of Intracellular Hydrogen Peroxide (H_2_O_2_) Levels

Because the change in the redox balance is a well-known phenomenon associated with particle exposure, we determined the intracellular hydrogen peroxide (H_2_O_2_) levels by several assays, i.e., the Peroxy-orange 1 (PO-1) fluorescent probe (Sigma-Aldrich, Darmstadt, Germany) and the OxiselectTM Hydrogen Peroxide/Peroxidase Assay Kit (STA-344—Cell Biolabs, Bio-connect, Huissen, The Netherlands).

**Figure A1:**
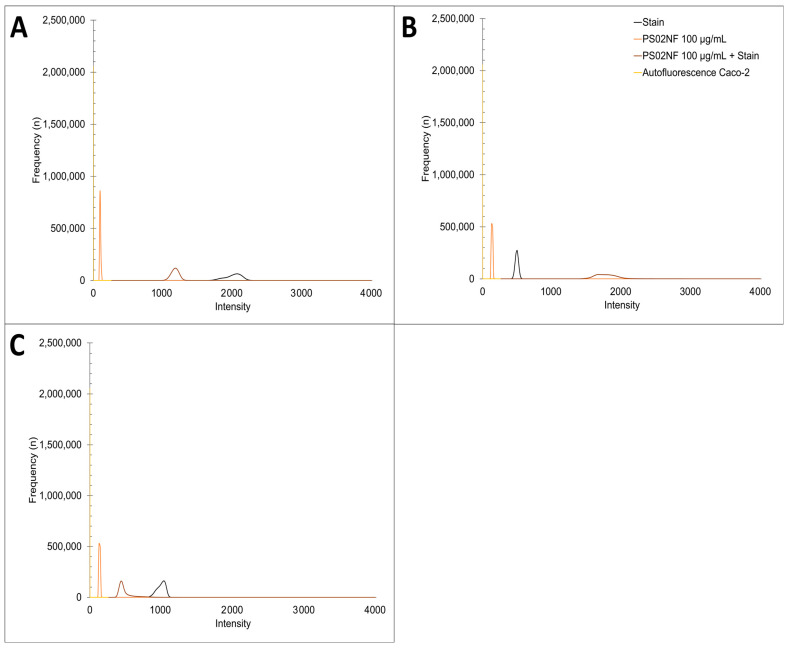
Autofluorescence of Caco-2 monolayer and particle interference with stain. Fluorescence intensities of stain in phosphate-buffered saline (PBS), PS02NF 100 µg/mL in PBS, PS02NF 100 µg/mL with stain in PBS, and caco-2 monolayer for (**A**) Peroxy-orange 1 (PO-1) (**B**) MitoTracker Red CMXRos and (**C**) MitoSOX were assessed with confocal laser scanning microscopy (LSM900, Zeiss, Zaventem, Belgium) and ZEN Blue 3.1 software (Zeiss, Zaventem, Belgium) at Ex/Em: 543/545–750.

PO-1 live staining was carried out on 3 × 10^4^ Caco-2 cells seeded in 8-well glass-bottom IBIDI chambers (Ibidi GmbH, Gräfelfing, Germany), grown in DMEM medium with FBS for 4–5 days at 5% CO_2_ and 37 °C until they reached 90–100% confluence. After 24 h of exposure to 0, 10, and 100 µg/mL of PS200NF and PS02NF, the cells were washed twice with PBS (+Ca^2+^, +Mg^2+^, 37 °C) and incubated for 40 min with 5 µM PO-1 staining solution at 37 °C and 5% CO_2_. Next, 20 min before the end, 300 µM H_2_O_2_ (Fisher Scientific, Brussels, Belgium) was added to a well to serve as a positive control. After incubation, the cells were rinsed twice with prewarmed PBS and immediately imaged with CLSM (LSM900, Zeiss, Zaventem, Belgium) at 10× magnification and Ex/Em: 543/545–750. Two tile scans (1.79 mm × 1.79 mm) were recorded for each condition with the ZEN blue 3.1 software (Zeiss, Zaventem, Belgium). The mean fluorescence intensity (MFI) ± standard error to the mean (SEM) was calculated by the random selection of 5 circles (circle diameter: 504.2 µm) on each image. In addition, the cells were also visualised using the same experimental setup without staining solution in order to detect possible autofluorescence (Figure A1A). Non-fluorescent particles were also tested for possible interference with PO-1 dye by measuring the MFI (Ex/Em: 543/545–750) of (i) PO-1 staining in PBS, (ii) non-fluorescent particles in PBS, and (iii) non-fluorescent particles in PO-1 staining in PBS (Figure A1A).

The Oxiselect^TM^ hydrogen peroxide/peroxidase assay was assessed on 6 × 10^4^ Caco-2 cells seeded in a 24-well culture plate and grown in DMEM medium with FBS for 4–5 days at 5% CO_2_ and 37 °C until they reached 90–100% confluence. After washing twice with PBS (+Ca^2+^, +Mg^2+^), the cells were exposed to 0, 10, and 100 µg/mL of PS200NF, PS02NF, and PSMF for 24 h in DMEM medium without FBS at 37 °C and 5% CO_2_. After exposure, the cells were washed twice with PBS (−Ca^2+^, −Mg^2+^), trypsinised, resuspended in DMEM medium with FBS, and centrifuged at 300× *g* for 5 min. The pellet was resuspended in 70 µL of 1× Assay buffer and 3–5 glass beads were added to disrupt it for 1 min at 30 Hz (Retsch Mixer Mill MM400, Fisher Scientific, Brussels, Belgium). Subsequently, 50 µL of each sample and standard were transferred to a black 96-well microplate (Greiner, Sigma-Aldrich, Darmstadt, Germany), followed by the addition of 50 µL of the Oxiselect^TM^ acetyl-3, 7-dihydroxyphenoxazine/horseradish peroxidase (ADHP/HRP) working solution to each well. The fluorescence intensity was measured using a microplate reader (Ex/Em: 544 nm/590 nm) (Fluostar Omega, BMG Labtech, Champigny-sur-Marne, France). To normalise the data, the total protein content was determined using the Bio-Rad protein assay (Bio-Rad Laboratories, Temse, Belgium) according to the manufacturer’s instructions.

#### 2.7.4. Measurement of Mitochondrial DNA Content

As the mitochondrial DNA fluctuates in response to the cellular environment, we determined the mitochondrial DNA content (mtDNAc) using RT-qPCR. Caco-2 cells were seeded at 6 × 10^4^ cells/24-well and grown to a confluent monolayer (5 days). After exposure to 0, 10, and 100 µg/mL PS200RF, PS02RF, and PSMF for 24 h in DMEM medium without FBS, the cells were washed in PBS, detached with 0.05% trypsin, and pelleted by centrifugation (300× *g*, 5 min, RT). After removing the supernatant, the cells were immediately frozen in liquid nitrogen and stored at −80 °C. DNA was isolated from the cells using phenol-chloroform extraction. The purity and concentration of the sample were determined using Nanodrop spectrophotometer (ND-1000, Fisher Scientific, Brussels, Belgium). To ensure a consistent DNA input of 5 ng for each RT-qPCR reaction, the samples were diluted and checked using the Quant-iT™ PicoGreen^®^ dsDNA Assay Kit (Life Technologies, Fisher Scientific, Brussels, Belgium). The relative mtDNAc was measured in triplicate using a previously described quantitative real-time PCR assay with minor modifications [28]. All reactions were performed on a 7900HT Fast Real-Time PCR System (Applied Biosystems, Fisher Scientific, Brussels, Belgium) in a 384-well format. Table 3 lists the mtDNA and single copy-genes that were used. The reaction mixtures contained Qiagen 1× QuantiTect SYBR Green master mix (Qiagen Benelux, Venlo, The Netherlands), forward primer, reverse primer, and 5 ng DNA. The thermal cycling profile was: 1 cycle of 10 min at 95 °C, followed by 40 cycles of 15 s at 95 °C, and 1 min 10 s at 58 °C. The raw data were processed and normalized to the single copy-genes using the 2^–∆∆Ct^ method. MtDNAc was expressed as the ratio of the mtDNA copy number to single-copy gene number (M/S) relative to the control.

#### 2.7.5. Measurement of Mitochondrial Superoxide Levels

The mitochondrial superoxide levels were measured as a marker for mitochondrial oxidative stress. The MitoSOX Red stain (2.5 µM, Invitrogen, Fisher Scientific, Brussels, Belgium) was loaded onto a confluent Caco-2 monolayer grown in an 8-well IBIDI chamber that was treated with 0, 10m and 100 µg/mL PS200NF for 24 h. After removing the culture medium and washing twice with PBS (+Ca^2+^, +Mg^2+^, 37 °C), 300 µL of MitoSOX Red stain (2.5 µM diluted in prewarmed PBS) was applied for 10 min (dark, 37 °C, 5% CO_2_). After rinsing twice with PBS, live imaging was performed using CLSM (LSM900, Zeiss, Zaventem, Belgium) at 40× magnification and Ex/Em: 510/580. For each condition, 2 tile scans (300 µm × 600 µm) were recorded using the ZEN blue 3.1 software (Zeiss, Zaventem, Belgium). The MFI ± SEM was calculated by randomly selecting 5 circles (circle diameter = 100 µm) for each image. The MFI shows mitochondrial superoxide production because the dye selectively targets the mitochondria in living cells, and it is rapidly oxidized by superoxide and not by other reactive oxygen or nitrogen species. In addition, both non-fluorescent particles and cells were also visualised using the same experimental setup without the staining solution to detect possible autofluorescence (Figure A1B). The possible interference of non-fluorescent particles with the MitoSOX Red dye was also checked (Figure A1B).

#### 2.7.6. Analysis of Mitochondrial Network Morphology

Because exposure to environmental stressors might affect mitochondrial functioning, we assessed the mitochondrial network morphology. After 24 h of exposure of the Caco-2 monolayer to 0, 10, and 100 µg/mL PS200NF in 8-well IBIDI chambers, the culture medium was removed and washed twice with prewarmed PBS (+Ca^2+^, +Mg^2+^). Then, 300 µL of MitoTracker Red CMXRos (250 nM, Invitrogen, Fisher Scientific, Brussels, Belgium), diluted in prewarmed PBS, was incubated on the cells in the incubator (dark, 37 °C, 5% CO_2_) for 45 min, followed by 2 washes in prewarmed PBS. Live imaging was immediately performed using CLSM (LSM900, Zeiss, Zaventem, Belgium) at 40× magnification and Ex/Em: 579/599. For each condition, a tile scan (300 µm × 600 µm) was recorded using the ZEN blue 3.1 software (Zeiss, Zaventem, Belgium). Based on the binary morphological skeleton obtained in ImageJ using the MiNA tool as described by Valente et al. (2017) [29], the mitochondrial footprint, branch length mean, and network branches mean were calculated. The mitochondrial footprint represents the area of the image consumed by the mitochondrial signal. The branch length mean is the average length of all the lines used to depict the mitochondrial structures. The network branches mean is the average number of confirmed lines to represent each network structure. Additionally, both non-fluorescent particles and cells were also visualised using the same experimental setup without staining solution in order to detect possible autofluorescence (Figure A1C). A possible interference of non-fluorescent particles with the MitoTracker Red CMXRos dye was also tested (Figure A1C).

#### 2.7.7. Statistical Analysis

All cellular assays were performed in triplicate and repeated twice in independent experiments unless stated otherwise. The results are presented as the mean ± standard deviation (SD) or the standard error of the mean (SEM) relative to the control. Gene expression was carried out in 3 independent experiments with 6 biological replicates and the results were presented as the mean ± SEM relative to the control. All statistical analyses were performed using the JMP^®^ Pro 16 (JMP Benelux, Medmenham Marlow, UK) software. The normality and homoscedasticity were tested with the Shapiro-Wilk and Bartlett’s tests, respectively. Normally distributed data were statistically tested with a one-way analysis of the variance (ANOVA) with Tukey’s or Dunnett’s post hoc tests to assess significant differences between the control and treatment groups. If the assumptions of normality and homoscedasticity were not satisfied after log or square root transformations, a non-parametric multiple comparison procedure using the Dunn method was performed. A *p*-value < 0.05 was considered statistically significant. For the gene expression data, agglomerative hierarchical clustering (based on raw Ct values) was performed with the free PAST software (version 4.03, developed by Oyvind Hammer, University of Oslo) using the UPGMA method computed with Euclidean distances. The cophenetic correlation coefficient was calculated to assess the significance of each dendrogram developed.

## 3. Results

### 3.1. Physical Characterisation of PS-MPs in Stock Suspension and Cell Culture Medium

**Figure 1:**
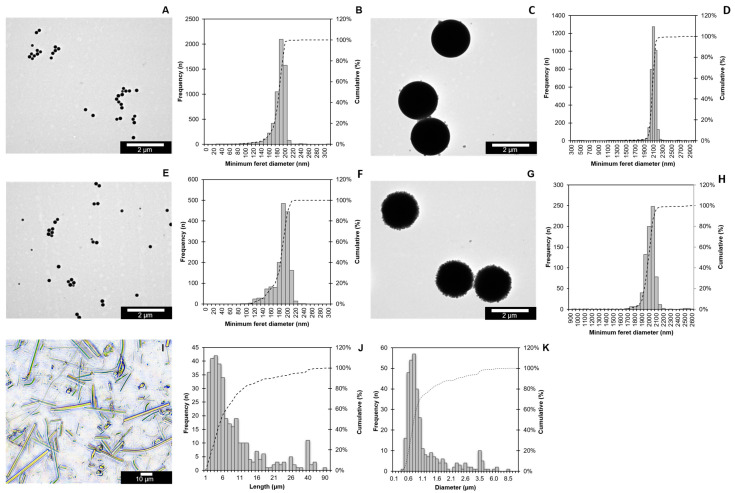
Size distribution of PS-MPs. Representative images of PS200RF (**A**), PS02RF (**C**), PS200NF (**E**), and PS02NF (**G**) were acquired by TEM, while a Nikon light microscope was used to visualise PSMF (**I**). The size distributions of the PS200RF (*n* = 5748) (**B**), PS02RF (*n* = 3490) (**D**), PS200NF (*n* = 1630) (**F**), and PS02NF (*n* = 743) (**H**) were assessed by the NanoDefine ParticleSizer plugin software in ImageJ 2.9.0/1.53t (NanoDefine 2016). Length (**J**) and Diameter (**K**) of PSMFs were measured manually using ImageJ (*n* = 350). Bars represent the frequency (*n*) (left *y*-axis), and dotted lines represent the cumulative frequency percentage (%) (right *y*-axis).

Image analysis of the TEM images showed a minimum Feret diameter ± SD of 0.180 µm ± 0.017 µm for the PS200RF and 2.073 µm ± 0.132 µm for the PS02RF, indicating a spherical shape (Figure 1A–D). Their non-fluorescently labelled counterparts, PS200NF and PS02NF, showed minimum Feret diameters (SDs) of 0.182 ± 0.021 µm and 1.988 ± 0.096 µm, respectively (Figure 1E–H). The DLS measurements showed that the hydrodynamic diameter of PS200RF and PS200NF stock suspensions were in the same size range (PS200RF: 0.216 µm and PS200NF: 0.225 µm). The surface zeta potential ± SD of PS200RF and PS200NF showed a negative surface charge in MilliQ of −40.39 mV ± 0.34 and −41.6 mV ± 0.48, respectively (Table 4). The SLS measurements indicated a hydrodynamic diameter (Dv50) of 2.14 µm for the PS02RF stock suspension, while the hydrodynamic diameter (Dv50) of the PS02NF stock suspension was 2.01 µm. When placed in the cell culture medium for 24 h, the hydrodynamic diameter changed for all PS-MPs, showing, in general, a larger hydrodynamic diameter. This increase in size is expected, owing to the adsorption of the biomolecules from the medium onto the particle surface. Based on light microscopy, the PSMF diameter and length ± SD averaged 1.14 µm ± 0.97 µm and 8.9 µm ± 10.1 µm, respectively, with both fibre-like and fragment-like shapes (Figure 1I–K).

### 3.2. Cytotoxicity Effects of PS-MPs in Caco-2 Cells

**Figure 2:**
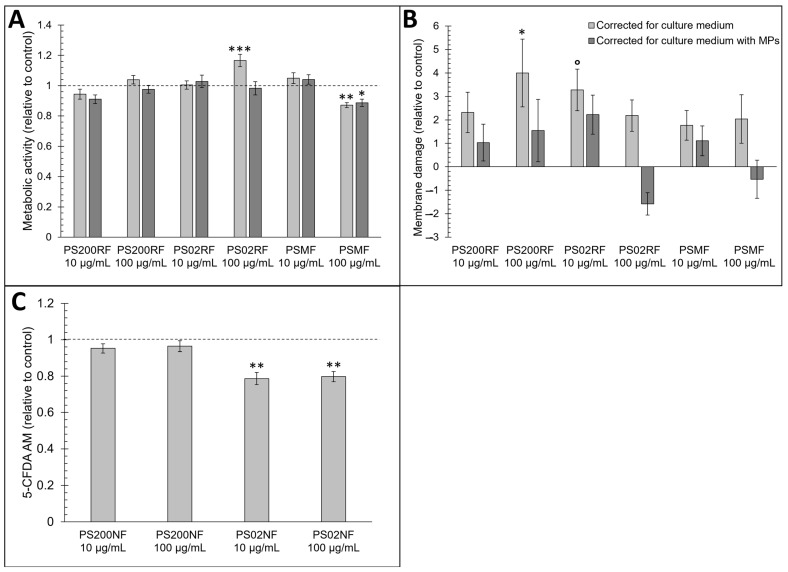
Cytotoxicity effects of PS200RF, PS02RF, and PSMF in a Caco-2 monolayer. (**A**) Metabolic activity determined with 3-(4,5-dimethylthiazol-2-yl)-2,5-diphenyltetrazolium bromide) tetrazolium reduction (MTT) assay. Data represent percentage metabolic activity ± standard error to the mean (SEM) relative to control (*n* = 9 of 3 independent experiments). Statistical significance was assessed by one-way ANOVA with control using Dunnett’s method. * *p* < 0.05, ** *p* < 0.01, *** *p* < 0.001. (**B**) Membrane damage determined by lactate dehydrogenase (LDH) assay. Data are presented as percentage membrane damage ± SEM (*n* = 6 from 2 independent experiments). Statistical significance was assessed by one-way ANOVA with control using Dunnett’s method. ° *p* < 0.1, * *p* < 0.05. (**C**) Membrane integrity determined with 5-carboxyfluorescein diacetate—acetoxymethyl ester (5-CFDA-AM) assay. Data are presented as percentage membrane integrity ± SEM relative to control (*n* = 4 from 4 independent experiments). Statistical significance was assessed with a nonparametric comparison with control using Dunn method for joint ranking. ** *p* < 0.01.

**Figure A2:**
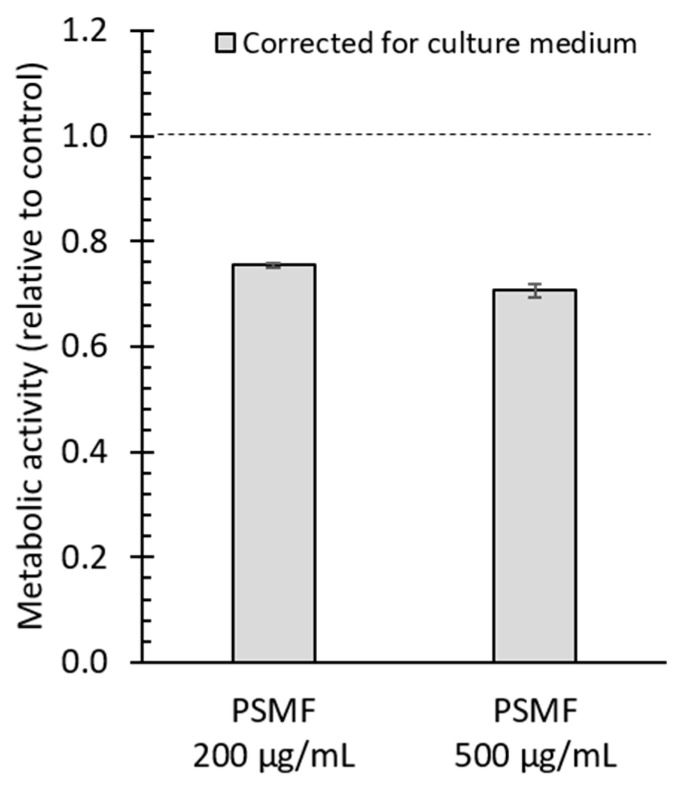
Cytotoxicity effects of PSMF. Metabolic activity (relative to control), measured with the 3-(4,5-dimethylthiazol-2-yl)-2,5-diphenyltetrazolium bromide) tetrazolium reduction (MTT) assay, of the Caco-2 monolayer after exposure to 0, 200, and 500 µg/mL PSMF (*n* = 4).

Small changes in metabolic activity were observed after 24 h of exposure to all PS-MPs (Figure 2A–C). Based on the MTT assay, a higher metabolic activity was observed after exposure to 100 µg/mL PS02RF (mean ± SEM: 116.6% ± 4.1%) after correction for the culture medium but disappeared after correction for condition-specific medium (mean ± SEM: 95.3% ± 4.4%) (Figure 2A). The correction for the condition-specific medium (Figure 2A) changed the metabolic activity for all PS-MPs, indicating that PS-MPs interfere with the optical readout signal. A lower metabolic activity was observed after exposure to 100 µg/mL PSMF (mean ± SEM: 87% ± 1.7%) compared to the control (Figure 2A), which was more pronounced at higher PSMF concentrations (mean ± SEM for 200 µg/mL: 75.4% ± 0.55%; and 500 µg/mL: 70.1% ± 1.3%) (Figure A2).

The membrane integrity of the Caco-2 monolayer was affected by two micron PS spheres. Based on the LDH assay, PS02RF (10 µg/mL) caused 3.27 ± 0.88% more membrane damage compared to the control but optical particle interference was observed that attenuated the effect (Figure 2B). For 5-CFDA-AM, no optical particle interference was detected. Both 10 and 100 µg/mL PS02NF showed a decreased membrane integrity (average ± SEM: 21.4 ± 3.3% and 21.3 ± 2.9%, respectively) (Figure 2C). Altogether, these findings indicate a subcytotoxic response following exposure to high concentrations of PSMF and PS02NF.

### 3.3. Association and Uptake of PS-MPs in a Caco-2 Monolayer

**Figure 3:**
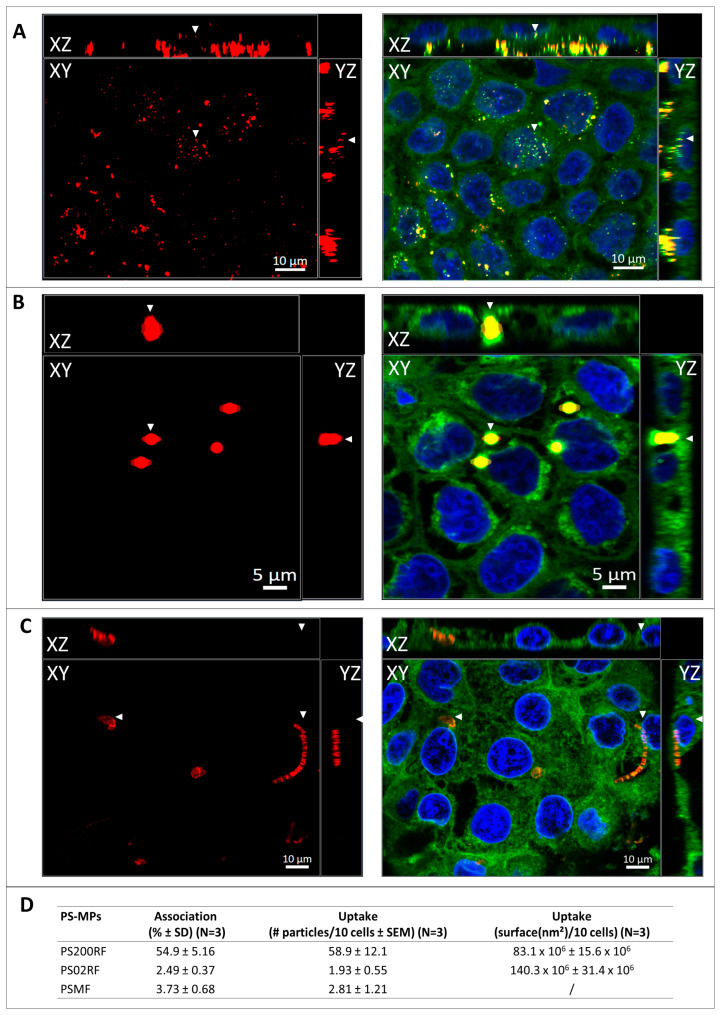
Association and uptake of PS-MPs in a Caco-2 monolayer. Representative XYZ-image of (**A**) PS200RF (Red/Yellow), (**B**) PS02RF (Red/Yellow), and (**C**) PSMF (Red) after 24 h of exposure to 100 µg/mL in a confluent Caco-2 monolayer (Nuclei—Hoechst 33342; Plasma membrane—CellMask Green Plasma Membrane stain). Fixed points of XYZ-images are indicated with arrowheads. (**D**) Semi-quantitative data of association (based on fluorescence-activated cell sorting (FACS), *n* = 3/condition) and uptake (based on confocal laser scanning microscopy (CLSM), *n* = 3 z-stacks/condition) of PS-MPs. Note: SD = standard deviation, SEM = standard error to the mean.

Using flow cytometry, we observed that 54.9 ± 5.16% of the Caco-2 cells were associated with PS200RF particles, while only 2.49 ± 0.37% and 3.73 ± 0.68% cells were associated with PS02RF and PSMF particles, respectively (Figure 3D). To further distinguish if the particles were taken up by the cells, we used confocal microscopy. XYZ-images showed that PS200RF and PS02RF particles were taken up by the Caco-2 monolayer after an exposure of 24 h (Figure 3A,B). Fibre- and fragment-like MPs with an average ± SD (*n* = 11) diameter of 2.52 ± 1.34 µm and length of 15.66 ± 9.96 µm were (partly) taken up (2.81 ± 1.21 particles/10 cells) (Figure 3C). A semi-quantitative assessment also showed that more PS200RF were taken up compared to PS02RF (58.9 ± 12.1 particles/10 cells versus 1.93 ± 0.55 particles/10 cells, respectively) (Figure 3D). It should be noted that the area covered by PS200RF in the cells is significantly smaller compared to that of PS02RF (83.1 × 10^6^ ± 15.6 × 10^6^ nm^2^/10 cells versus 140.3 × 10^6^ ± 31.4 × 10^6^ nm^2^/10 cells) (Figure 3D).

### 3.4. Effects of PS-MPs Exposure on the Oxidative Stress Response in a Caco-2 Monolayer

**Figure 4:**
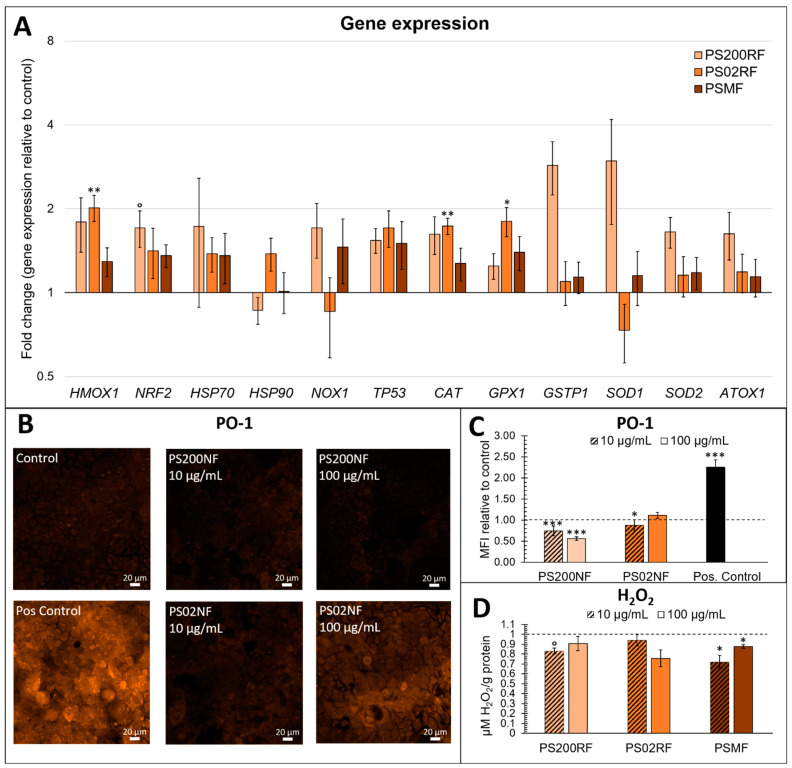
Effects of PS-MPs on oxidative stress responses in a Caco-2 monolayer. (**A**) Gene expression of oxidative stress-related genes after 24 h of exposure to 0 and 100 µg/mL PS200RF, PS02RF, and PSMF in a Caco-2 monolayer. Results represent the fold change expression to control ± standard error to the mean (SEM) (above 1 means up-regulated, below 1 means down-regulated). The fold changes of genes were normalised using three reference genes (Beta-2 microglobulin, Ribosomal protein P0, and Ubiquitin C). *n* = 10–17 from three independent experiments. One-way ANOVA with control using Dunnett’s method or nonparametric comparison with control using Dunn method for joint ranking was used to assess significance. ° *p* < 0.1, * *p* < 0.05, ** *p* < 0.01. (**B**) Representative confocal laser scanning microscopy (CLSM) images of hydrogen peroxide (H_2_O_2_) anions detected in a Caco-2 monolayer after 24 h of exposure to 0, 10, and 100 µg/mL PS200NF and PS02NF using Peroxy-orange 1 (PO-1) live staining on CLSM. Addition of 300 µM H_2_O_2_ on the Caco-2 monolayer was used as positive control. (**C**) Fluorescence intensity of H_2_O_2_ anions based on PO-1 staining recorded by CLSM at 20× magnification. Results represent mean fluorescence intensity (MFI) ± SEM from 2 independent experiments (*n* = 5 random circles of ±500 µm diameter/image based on 2 images per condition). Statistical significance was assessed by one-way ANOVA with control using Dunnett’s method. * *p* < 0.05, *** *p* < 0.001. (**D**) H_2_O_2_ levels detected in a Caco-2 monolayer after 24 h of exposure to 0, 10, and 100 µg/mL PS200NF, PS02NF, and PSMF based on quantitative 10-Acetyl-3, 7-dihydroxyphenoxazine/horseradish peroxidase (ADHP/HRP)-based assay. Results represent average H_2_O_2_ levels (µM/g protein) ± SEM relative to control (*n* = 6 from 2 independent experiments). Nonparametric comparison with control using Dunn method for joint ranking was used to assess statistical significance. ° *p* < 0.1, * *p* < 0.05.

**Figure 5:**
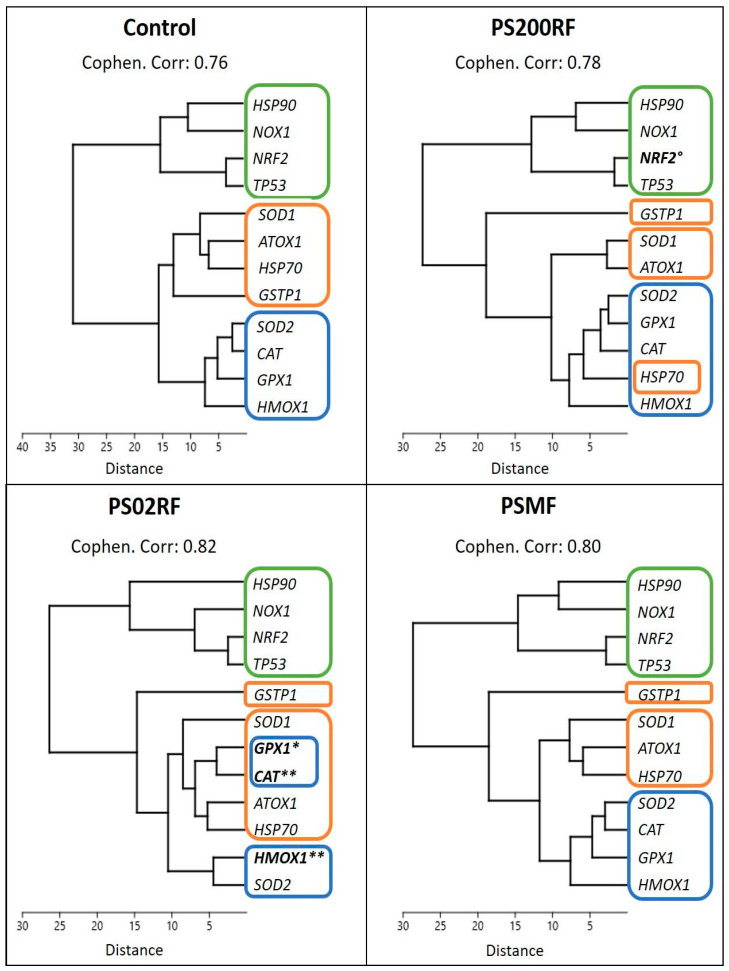
Hierarchical clustering of gene expression results per treatment group. Unweighted pair group method with arithmetic mean (UPGMA) with Euclidean distance was used to construct the dendrogram. Cophenetic correlation coefficient was calculated to measure the significance of the clustering process. Each coloured rectangle represents a cluster with the control condition as baseline. Gene names in bold were statistically significant compared to control (*n* = 10–17 from 3 independent experiments) (based on Figure 4A).

The gene expression analysis of redox-related genes mainly showed differential gene expression patterns after exposure to PS02RF (100 µg/mL) compared to the unexposed cells (Figure 4A). Significant changes were observed for *HMOX1* (Fold Change (FC): 2.09 ± 0.32, *p* = 0.0042), *CAT* (FC: 1.95 ± 0.19, *p* = 0.0072), and *GPX1* (FC: 1.65 ± 0.19, *p* = 0.023). Following PS200RF exposure, only *NRF2* was differentially expressed (FC: 1.71 ± 0.26, *p* = 0.058), while PSMF exposure did not alter the gene expression significantly (Figure 4A). Hierarchical clustering of the genes further revealed that genes co-expressed within the control group were also similarly co-expressed after PSMF exposure (Figure 5), while for PS200RF and PS02RF exposure, the *HSP70* gene and *CAT, GPX1* genes, respectively, changed from co-expression network, as compared to the control group (Figure 5). We further assessed the H_2_O_2_ levels qualitatively and quantitatively by PO-1 staining and an ADHP-based H_2_O_2_ assay, respectively. Both assays showed lower intracellular H_2_O_2_ levels for the PS spheres compared to the unexposed cells (Figure 4B–D), with the most prominent changes occurring for the PS200NF. On average ± SEM 25 ± 11% (*p* = 0.0003) and 44 ± 4% (*p* < 0.0001) lower H_2_O_2_ levels (based on PO-1 staining) were observed for the 10 and 100 µg/mL PS200NF, respectively, as well as for the 10 µg/mL PS02RF (12 ± 13%, *p* = 0.02) (Figure 4B,C). For the ADHP-based H_2_O_2_ assay, lower H_2_O_2_ levels were observed for PS200RF (10 µg/mL: −17 ± 7%; *p*= 0.03) and PSMF exposure (10 µg/mL: −28 ± 7%, *p* = 0.004; 100 µg/mL: −12 ± 2%, *p* = 0.03) compared to the control (Figure 4D). 

### 3.5. Effects of PS-MPs on Mitochondrial Functioning in a Caco-2 Monolayer

**Figure 6:**
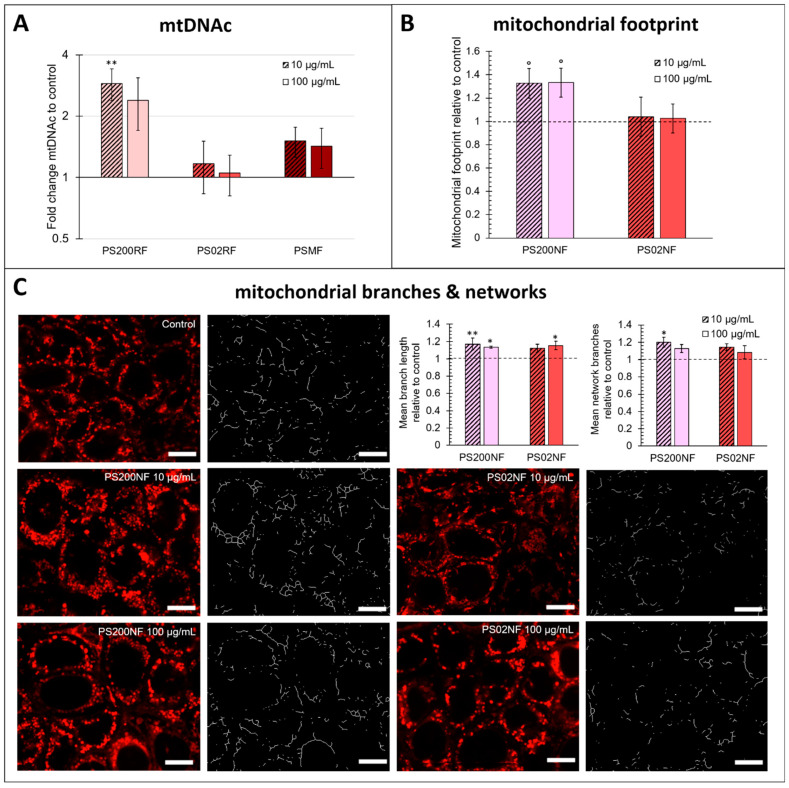
Effects of PS-MPs on mitochondrial functioning in a Caco-2 monolayer. (**A**) Mitochondrial DNA content (mtDNAc) (fold change to control) of a Caco-2 monolayer after 24 h of exposure to 10 and 100 µg/mL PS200RF, PS02RF, and PSMF (*n* = 12 from 2 independent experiments). (**B**) Mitochondrial footprint (relative to control) of a Caco-2 monolayer after 24 h of exposure to 10 and 100 µg/mL PS200NF and PS02NF (*n* = 6 from 2 independent experiments). (**C**) Representative images of the different conditions with MitoTracker Red CMXRos and their corresponding skeleton assessed by the MINA tool in ImageJ according to Valente et al. 2017 [29]. Scale bar: 10 µm. The graphs represent the mean branch length and mean branching networks (relative to control) (*n* = 6 from 2 independent experiments) of a Caco-2 monolayer after 24 h of exposure to 0, 10, and 100 µg/mL PS200NF and PS02NF. Statistical significance for all results was assessed using one-way ANOVA with control using Dunnett’s method. ° *p* < 0.1, * *p* < 0.05, ** *p* < 0.01.

**Figure A3:**
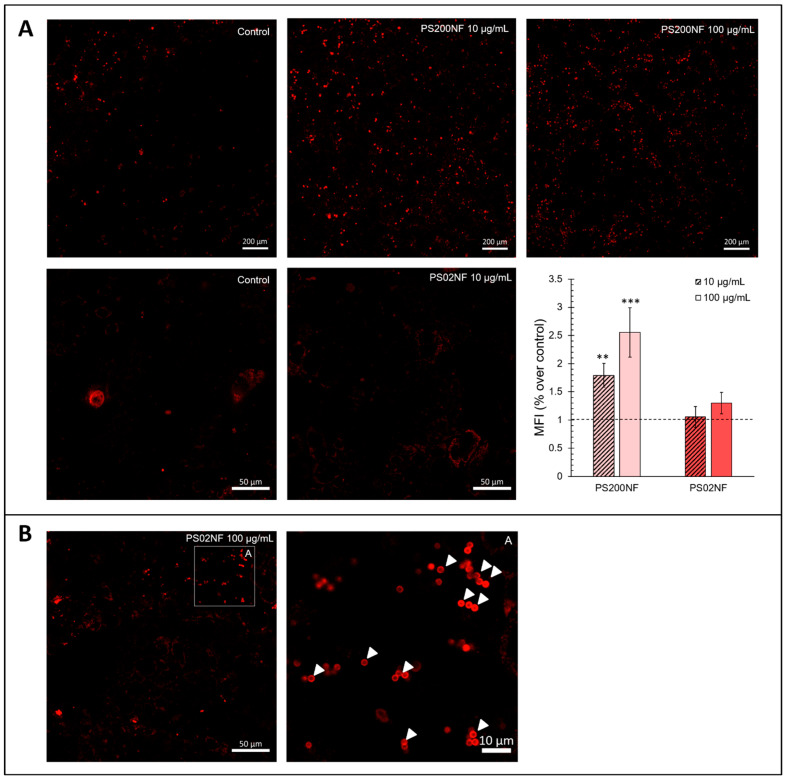
Mitochondrial superoxide levels of a Caco-2 monolayer after 24 h of exposure to PS200NF and PS02NF. (**A**) Representative confocal laser scanning microscopy (CLSM) images of mitochondrial superoxide anions detected in a Caco-2 monolayer after 24 h of exposure to 0, 10, and 100 µg/mL PS200NF and PS02NF using mitochondrial superoxide (MitoSOX) live staining. Results represent the mean fluorescence intensity (MFI) ± standard error to the mean (SEM) relative to control from 2 independent experiments (*n* = 5 random circles of ± 500 µm diameter/image based on 2 images per condition). Statistical significance was assessed by one-way ANOVA with control using Dunnett’s method. ** *p* < 0.01, *** *p* < 0.001. (**B**) Representative image of PS02NF (indicated by white arrows) stained with MitoSOX during cell experiments.

MtDNAc was higher compared to the control after exposure to PS200RF with a significant fold change difference of 2.89 ± 0.51 (*p* = 0.005) for 10 µg/mL exposure (Figure 6A). No changes in mtDNAc were found after exposure to PS02RF or PSMF. These results were supported by the larger mitochondrial footprint under PS200NF exposure compared to the unexposed cells (10 µg/mL: +32.7 ± 12.7%, *p* = 0.07; 100 µg/mL: +33.3 ± 12.3%, *p* = 0.07) (Figure 6B). In addition, changes in the mitochondrial morphology were observed, especially for the PS200NF spheres. The mean mitochondrial branch length (10 µg/mL: +17.2 ± 7%, *p* = 0.007; 100 µg/mL: +13.4 ± 1.3%, *p* = 0.04) and the mean number of mitochondrial network branches (10 µg/mL: +20.0 ± 5.6%, *p* = 0.01) were more present after exposure to PS200NF compared to the controls (Figure 6C). For PS02NF, the results were less pronounced with only a significantly higher mean mitochondrial branch length after exposure to 100 µg/mL (+15.4 ± 4.7%, *p* = 0.02). Finally, the mitochondrial superoxide levels were also significantly increased after exposure to PS200NF (10 µg/mL: +79.1 ± 21.4%, *p* = 0.005; 100 µg/mL: 255 ± 44%, *p* < 0.0001) (Figure A3A), but these results should be interpreted with caution as we also observed a fluorescent signal when we combined the non-fluorescent particles with the stain in a set-up without cells (Figure A3B).

## 4. Discussion

Microplastics (MPs) are a major concern for both the (marine) environment and human health. MPs were found in human stool [13], blood [14], and placental tissue [15], but the associated health risks are unknown. To date, most in vitro studies use commercially available MP beads, which do not fully reflect real-life exposure. Fibrous and fragmented MPs account for more than 50% of the MPs present in the environment. To fill this existing research gap, our study included both spherical-, fibre-, and fragment-shaped PS-MPs and evaluated their uptake profiles and intracellular effects in an in vitro intestinal model system to meet the 3R requirements. The key findings of this study will help us understand the toxicity of MPs of different sizes and shapes, a necessity emphasised by SAPEA, a consortium of academic networks that is part of the European Commission’s Scientific Advice Mechanism, in their report of 2019 [23] and by the WHO in 2022 [30].

Our study found that spherical PS-MPs of 200 nm and 2 µm, as well as a combination of fibre- and fragment-shaped PS-MPs, were taken up by Caco-2 cells. Most previous studies investigated the uptake of mainly spherical micro- and nanoplastics, as shown in a recent review [22]. For a wide variety of cell types, it was found that spherical particles ranging from 25 nm to 10 µm were internalised, which is consistent with our findings for PS 200 nm and 2 µm spheres. Our semi-quantitative data showed stronger uptake of the smaller PS 200 nm spheres compared to the PS 2 µm spheres, although the area covered by the PS 200 nm spheres in the cells is smaller than that of the PS 02 µm spheres. A higher uptake of the smaller particles was also observed by Wu et al. (2019) using PS 100 nm and 5 µm spheres after 12 h exposure in Caco-2 cells [31]. Wang et al. (2020) assessed the cellular uptake of micro-sized PS spheres (300 nm to 6 µm, 20 µg/mL, 24 h) in Caco-2 cells and reported uptake levels ranging from 73% to 30%, respectively, indicating that the internalisation of smaller particles is favoured [32]. However, Sendra et al. (2020) reported that in bivalve granulocytes a correction for the number of particles exposed to the cells did not necessarily favour uptake of smaller sizes (without correction: 89.7 ± 1.9%, 60.6 ± 3.5%, and 59.3 ± 5.3% uptake; with correction: 0.5 ± 0.1%, 13.9 ± 27.4%, and 52.5 ± 7.1%, for, respectively, 50 nm, 100 nm, and 1 µm PS spheres) [33]. Hence, variations in the particle number for the same exposure dose should be taken into account as these might explain the differences in the particle uptake. In our study, we used the same mass concentrations for the different sizes of PS spheres. This corresponded to a 1000× higher number of particles for the PS 200 nm (100 µg/mL: 2.18 × 10^10^ particles/mL) compared to the PS 2 µm spheres (100 µg/mL: 2.26 × 10^7^ particles/mL). Another explanation for the differences in particle uptake is the different uptake mechanisms that are involved in the uptake of sub-micron and micron-sized PS spheres. A recent review by Manzanares and Cena (2020) emphasised that, although other pathways can be involved, receptor-mediated endocytosis is the most prominent uptake mechanism of sub-micron particles with an upper size limit of 150–200 nm [34]. The uptake of larger particles generally depends on phagocytosis [34]. Apart from size, the mechanism of uptake also depends on the state of agglomeration, the substances adsorbed on the surface, and the surface charge of the particles when exposed to cells in the culture medium. With respect to the fibre-/fragment-shaped MPs, our study showed that particles with diameters smaller than 2.52 µm and lengths smaller than 15.66 µm were taken up by a Caco-2 monolayer. Furthermore, our semi-quantitative data showed that the uptake of fibre-/fragment-shaped PS-MPs was comparable to that of the PS 2 µm spheres. The lack of studies on non-spherical MPs makes comparison with the literature difficult. Magri et al. (2018) found PET nano fragments (27 nm) inside Caco-2 cells [35], while Stock et al. (2021) observed no uptake of irregular PE (90.1 µm), PET (60 µm), or PVC (136.5 µm) [36]. These studies and our findings indicate that the fibre/fragment particle size plays an important role in cellular uptake profiles. This is consistent with the literature on spherical particles, where size is also presented as an important particle characteristic for the degree of cellular uptake [37,38].

Although higher uptake was observed for the PS 200 nm spheres compared to the PS 2 µm spheres and PSMFs, subcytotoxic effects in metabolic activity and membrane integrity were mainly found for PS 2 µm spheres and PSMFs. Based on the MTT assay, we did not detect changes in the metabolic activity of PS 200 nm and 2 µm spheres in Caco-2 cells after 24 h of exposure in medium without FBS (cell viability > 90% compared to controls), but mild effects were observed for PSMF, especially at exposure concentrations higher than 100 µg/mL (500 µg/mL: ± 70% compared to control condition). Based on the LDH and 5-CFDA AM assays, the membrane integrity was mainly affected by the PS 2 µm spheres; however, the cell viability was still >70% compared to the unexposed cells. Contradictory findings were reported regarding the cytotoxicity of MPs [18,35,39,40,41,42,43]. For example, Abdelkhaliq A et al. (2018) did not observe cytotoxicity in Caco-2 cells using the WST-1 assay after 24 h of exposure to PS carboxyl-modified 50 nm and 200 nm spheres [44]. Similar findings were reported by Cortes et al. (2020) [45] and Wu et al. (2019) [31] after exposure to PS unmodified 50 nm spheres using the Beckman counter method and PS unmodified 100 nm and 5 µm spheres using CCK-8 assay, respectively. Stock et al. (2019), however, observed cytotoxicity in Caco-2 cells using an MTT assay for 1 µm PS carboxyl-modified spheres at high concentrations (>1 × 10^7^ particles/mL) [18], and Choi D et al. (2020) found a reduction in viability after a one-day treatment with PS micro fragments (5–200 µm) at concentrations of up to 1000 µg/mL in immune-related cells using the CCK-8 assay [46]. We should be cautious when interpreting the results of standard cytotoxicity assays because recent research has shown that nanoparticles, such as AuNPs and AgNPs, cause particle interference (i.e., signal interference with the read-out signal). This can occur either through direct optical interference or through interaction with the assay components [47,48,49]. Therefore, we decided to use multiple assays in this study to fully investigate cytotoxicity and to screen for unintended particle contributions to the assay. Our results indicated optical interference of the polystyrene microplastics in absorbance-based assays (particles show absorbance at read-out wavelength) (Figure A1), which might explain the inconsistent findings in the literature by masking cytotoxicity. However, the common result in the three assays used was that mainly micron-sized particles showed subcytotoxic effects.

We further investigated the underlying redox-related signatures as reactive oxygen species (ROS) are often proposed as a mediator of particle-induced responses. We also looked at mitochondrial responses as mitochondria are a major source of ROS and actively participate in the regulation of cellular metabolisms [50]. At the gene expression level, the PS 2 µm spheres mainly induced changes in the transcriptional expression of oxidative- and anti-oxidative genes. Hierarchical clustering confirmed this result since the co-expression of the redox-related genes was found in similar networks for the PS 200 nm spheres and PSMF, but in slightly different networks for the PS 2 µm spheres. In particular, a higher expression of *HMOX1*, *CAT*, and *GPX1* was observed after exposure to PS 2 µm spheres, suggesting an important role of the heme oxygenase 1 (HO-1) system. Dong et al. (2020) [51] found an increased HO-1 protein expression after exposure to PS spheres (1.72 µm) in lung epithelial cells (BEAS-2B), especially at high concentrations (1000 µg/cm^2^) compared to low-dose concentrations (10 µg/cm^2^). The authors postulated that high exposure resulted in massive ROS generation that overcame the cellular anti-oxidative enzyme capacity, leading to oxidative stress, while low exposure resulted in a compensatory increase in HO-1 as an attempt to maintain the redox balance [51]. The latter could be one of the most important defensive responses against PS 2 µm spheres since the HO-1 system stimulates HO-1 expression as a cytoprotective effect to eliminate reactive-free heme-induced cytotoxicity. In addition, CAT and GPX which convert H_2_O_2_ to H_2_O in the mitochondria serve as the first line of antioxidant defence against ROS [52]. In this context, lower H_2_O_2_ levels were found after exposure to the lowest concentration of PS 2 µm spheres, but this observation was assay dependent. Overall, the altered intracellular redox balance could explain the subcytotoxic changes observed at the cellular level following PS 2 µm sphere exposure.

Although no significant gene expression changes were observed after exposure to both PS 200 nm spheres and PSMF, lower intracellular H_2_O_2_ levels and more mitochondria were detected as evidenced by a higher mtDNAc. It is well understood that mtDNAc fluctuates in response to the physiological environment surrounding the cell and might, therefore, reflect an altered cellular redox balance [53]. Exposure to PS 200 nm spheres resulted in significant alterations of different mitochondrial parameters, i.e., the number of mitochondria, the mean branch lengths, and the number of branched networks. Mitochondria undergo fusion and fission processes, resulting in fragmented states or continuous networks [29,54]. Changes in the mitochondrial morphology can have an impact on the mitochondrial function, leading to changes in energy production, a shift in the redox balance, and even cell death [54]. In this context, a recent study in HepG2 cells showed changes in mitochondrial dynamics and biogenesis after exposure to silver nanoparticles [55]. Cortes et al. (2020) and Wu et al. (2019) showed increased mitochondrial membrane potential and reduced ATP synthesis in Caco-2 cells after exposure to microplastics [31,44], respectively, which was also related to particle uptake [28]. We hypothesize that the high number of uptake of PS 200 nm spheres found in our study may play an important role in the redox and mitochondrial stress response in Caco-2 cells. A recent review highlighted that microplastics may decrease ATP content, reduce mitochondrial membrane potential and cause damage to the mitochondrial structure in human and other animal cells [56], supporting our hypothesis that the mitochondrial processes combined with redox processes are key in the defensive response towards microplastics. The higher mitochondrial superoxide levels observed in the current study after exposure to the PS 200 nm spheres support this hypothesis but should be interpreted with caution due to particle interference.

This study acknowledges both its strengths and limitations. First, particle interference with existing assays complicates experiments and result interpretation. In our research, we discovered that some absorbance-based assays are susceptible to interference caused by particles to the optical read-out. Furthermore, non-fluorescent PS 2 µm spheres became fluorescent in the standard assay, indicating interaction of the dyes on the surface of the PS spheres. This is plausible considering the surface charge density of the particles (Table 1). Whether this is also true for the PS 200 nm spheres was not examined in the present study. To fully assess toxicity responses and avoid misinterpretation, we used multiple assays and examined parameters at different biological levels. Second, several independent experiments were performed to account for the variability within and between experiments caused by the non-homogeneous distribution of particles in suspension. The use of multiple parameters to describe an outcome also contributed to the robustness of the described effects. Third, we did not use FBS in our experiments, which may contribute to the variation in certain effects. Fourth, we observed the uptake of different types of microplastics into the cells but where the microplastics accumulated in the cells was beyond the scope of this study. Finally, we used the particle mass to equate exposure doses for our experiments which might complicate determining the effects of size and shape on uptake at the single particle level. In addition to particle mass, future research should also consider particle size distributions and particle numbers as key characteristics for defining uptake and the underlying effects.

## 5. Conclusions

Our study revealed that, in addition to spherical PS-MPs, fibre- and fragment-shaped PS-MPs are also taken up by Caco-2 cells. Uptake clearly depends on the particle size. Although subcytotoxic changes were observed mainly for the micron-sized spherical and fibre-and fragment-shaped PS-MPs, a redox response was found to be induced for all the particles. This response varied in time and location, depending on the particle type, size, and number, with the PS 200 nm spheres having the most profound effects. Nonetheless, our results showed that the interaction between mitochondria and ROS, including the HO-1 system, are key regulators of the microplastic-induced defensive response in Caco-2 cells. Since the existence of other additional key factors cannot be ruled out, further research is needed to delve deeper into intracellular redox dynamics and to fully understand the underlying mechanisms of varying sizes, shapes, surface groups, and charges of MPs. In addition, it should be noted that particle interference in the assay results can lead to misinterpretation, underpinning the importance of measuring a combination of parameters for hazard profiling of MPs.

## Figures and Tables

**Table 1:** Specifications of commercially available PS beads provided by the manufacturer.

Characteristics	PS Spheres			
	PS200RF	PS200NF	PS02RF	PS02NF
Percentage solids (%)	2.5	10	2.5	10
Diameter (µm ± SD)	0.215 ± 0.022	0.217 ± 0.024	2.0 ± 0.1	2.0 ± 0.11
Polymer density (g/mL)	1.05	1.05	1.05	1.05
Surface groups	Carboxyl	Carboxyl	Carboxyl	Carboxyl
Parking area (A^2^/COOH group)	103	91.6	81.3	13
Surfactant	Anionic	Anionic	Anionic	Anionic
Crosslinking level (DVB/styrene, %*w*/*w*)	0	0	0.1	0.1
Preservative (sodium azide) (%)	0.09	0.09	0.09	0.09
Charge density (meq/g)	0.043	0.048	0.006	0.037
Fluorescent dye content (%*w*/*w*)	0.68%	-	0.3%	-

**Table 2:** Selection of genes and primer information.

Gene	Name	Accession Number(s)	Forward Primer (5′-3′)	Reverse Primer (5′-3′)
**Oxidative stress-related genes**				
*HMOX1*	Heme oxygenase 1	NM_002133.3	CTGCTCAACATCCAGCTCTTTG	CTCCACGGGGGCAGAATCTT
*NRF2*	Nuclear factor erythroid 2-related factor 2	NM_001313903.2	GATCTTGGAGTTGCCCACAT	ACGTAGCCGAAGAAACCTCA
*CAT*	Catalase	NM_001752.4	AGCTTAGCGTTCATCCGTGT	GCCACTAGCTTGCATTTG
*SOD1*	Superoxide dismutase 1	NM_000454.5	TGCAGGTCCTCACTTTAATCCTC	AGTCTCCAACATGCCTCTCTTC
*SOD2*	Superoxide dismutase 2	NM_001322819.2	AGCCCAGATAGCTCTTCAGC	CCAGCAACTCCCCTTTGGG
*GSTP1*	Glutathione S-transferase pi 1	NM_000852.4	TACACCAACTATGAGGCGGG	CAGCAGGTTGTAGTCAGCGA
*GPX1*	Glutathione peroxidase 1	NM_000581	TCCGGGACTACACCCAGATG	TCTTGGCGTTCTCCTGATGC
*ATOX1*	Antioxidant 1 copper chaperone	NM_004045.4	GTCCTCAATAAGCTTGGAGGAGTTA	AGGGTTGCAAGCAGAGTGTC
*NOX1*	NADPH oxidase 1, transcript variants 1, 2 and 3	NM_001271815.2 NM_013955.3 NM_007052.5	TTGGAGCAGGAATTGGGGTC	TTGAGGTTGTGGTCTGCACA
*HSP70*	Heat-shock protein family A (hsp70) member 1A, 1B and 8	NM_005345.6 NM_005346.6 NM_006597.6	CCGAGAAGGACGAGTTTGAG	CTGGTACAGTCCGCTGATGA
*HSP90*	Heat-shock protein 90 alpha member 1A, transcript variants 1 and 2	NM_001017963.3 NM_005348.4	AGACCCAGTCTTGTGGATGG	TACTCCCCTTTCCCCCTAAA
**Reference genes**				
*RPLP0*	Ribosomal protein P0	NM_001002.4	CGTCCTCGTGGAAGTGACAT	TAGTTGGACTTCCAGGTCGC
*B2M*	Beta-2-microglobulin	NM_004048.4	GATGAGTATGCCTGCCGTGT	CTGCTTACATGTCTCGATCCCA
*UBC*	Ubiquitin C	NM_021009.7	CAGCCGGGATTTGGGTCG	CACGAAGATCTGCATTGTCAAGT

**Table 3:** Primer information and input concentration of mtDNA and single-copy genes.

Gene	Forward Primer (5′-3′)	Reverse Primer (5′-3′)	Input Concentration
mtDNA * gene			
MT-ND1 *	ATGGCCAACCTCCTACTCCT	CTACAACGTTGGGGCCTTT	300 nM
Single copy-genes			
HBG1 *	GCTTCTGACACAACTGTGTTCACTAGC	CACCAACTTCATCCACGTTCACCA	400 nM
RPLP0 *	GGAATGTGGGCTTTGTGTTC	CCCAATTGTCCCCTTACCTT	400 nM

* mtDNA = mitochondrial DNA, *MT-ND1* = Mitochondrial encoded NADH dehydrogenase 1, *HBG1* = Haemoglobin Subunit Gamma 1, *RPLP0* = Ribosomal protein P0.

**Table 4:** Physical characteristics of PS-MPs based on TEM and DLS/SLS in stock suspension and cell culture medium.

Technique	Measurement	Medium	PS-MPs			
			PS200RF	PS200NF	PS02RF	PS02NF
TEM *	Feret min in µm (±SD) *	Stock	0.180 (±0.017)	0.182 (±0.021)	2.073 (±0.132)	1.988 (±0.096)
DLS *	Z-average in µm (PD ± SD) *	Stock	0.2165 (0.014 ± 0.017)	0.2247 (0.017 ± 0.019)	/	/
	Z-average in µm (PD ± SD) *	DMEM–FBS	0.2562 (0.052 ± 0.027)	0.2823 (0.083 ± 0.011)	/	/
	Zeta potential in mV (±SD) *	Stock	−40.39 (± 0.34)	−41.6 (± 0.48)	/	/
SLS *	Dv(10) − Dv(50) − Dv(90) in µm*	Stock	/	/	1.88 − 2.14 – 2.47	1.68 − 2.01 – 2.40
	Dv(10) − Dv(50) − Dv(90) in µm*	DMEM–FBS	/	/	1.80 − 2.20 – 2.69	1.58 − 2.08 – 2.73

* TEM = Transmission electron microscopy, DLS = Dynamic light scattering, SLS = Static light scattering, Feret min = minimum Feret diameter, Z-average = mean intensity-based diameter, PDI = polydispersity index where PDI < 0.05 (monodisperse) and PDI > 0.7 (broad size distribution), SD = standard deviation, Dv(10) = maximum particle diameter below which 10% of the sample volume exists, Dv(50) = median particle size by volume, Dv(90) = maximum particle diameter below which 90% of the sample volume exists.

## Data Availability

The datasets supporting the conclusions of this article can be retrieved from the corresponding author upon reasonable request.
